# Students’ perceived research skills development and satisfaction after completion of a mandatory research project: results from five cohorts of the Sydney medical program

**DOI:** 10.1186/s12909-023-04475-y

**Published:** 2023-07-12

**Authors:** Rajneesh Kaur, Jonathan Hakim, Richmond Jeremy, Genevieve Coorey, Eszter Kalman, Rebekah Jenkin, David G Bowen, Joanne Hart

**Affiliations:** 1 School of Medicine, Faculty of Medicine and Health, University of Sydney, Sydney, 2006 Australia; 2 School of Health Sciences, Faculty of Medicine and Health, University of Sydney, Sydney, 2006 Australia; 3 Office of the Deputy Vice Chancellor (Education), Educational Innovation Team, DVC(E) Portfolio, University of Sydney, Sydney, 2006 Australia; 4 School of Medical Sciences, Faculty of Medicine and Health, University of Sydney, Sydney, 2006 Australia

**Keywords:** Medical students, Research projects, Research skills, Student satisfaction

## Abstract

**Background:**

Research activities undertaken during University studies contribute to preparation of medical students for practice of evidence-based medicine. This study aimed to understand medical students’ experiences, perceived research skills development and satisfaction associated with completion of mandatory research projects.

**Methods:**

An online survey was sent to five cohorts of students (n = 1375) from years 2017–2021 at the completion of their research projects. Univariate analysis was conducted to understand students’ perception of research skills development, followed by linear regression modeling to explore factors influencing satisfaction with their research project. Manifest content analysis employing a framework approach was used to analyse qualitative data from responses to open ended questions.

**Results:**

Response rate was 42%, with 513 (89%) returned surveys being complete and included in analysis. Whilst 37% of students felt they had requisite research skills before undertaking the research project, 84% reported they had these skills after completing the project (χ^2^ = 8.99, P = 0.02). Mean satisfaction score of the students was 5.0/10 (+/- 2.5, median = 6 (IQR = 3.0–7.0) with 59% of students reporting satisfaction scores higher than the average. Higher satisfaction scores were reported by those who perceived that: research methods and teaching was useful in preparing them for conducting research; the research project helped them acquire new skills; the project resulted in peer-reviewed publication; and, who felt supported by their supervisors. Responses to open ended questions offered important insights into student experience and emphasised the importance of supportive supervisors and the need for a dedicated research block in the busy medical program.

**Conclusions:**

The majority of students reported positive outcomes from the mandatory research project. Student satisfaction can be improved by ensuring supportive research environments and high-quality supervision, and inclusion of dedicated research time in the medical curriculum.

**Supplementary Information:**

The online version contains supplementary material available at 10.1186/s12909-023-04475-y.

## Introduction

The practice of evidence based medicine (EBM) requires medical practitioners to acquire, appraise and apply the best research evidence to their clinical practice [1–5]. Research activities such as research projects undertaken during the medical degree, can assist to equip students with essential skills to practice EBM [1–5]. It is well documented that research projects provide medical students with key research skills and enhance their confidence to undertake research during their future clinical practice and professional career [6, 7]. Research activities undertaken during the medical degree also have the potential to encourage or deter future participation in research [8]. A poor research experience consequent upon a lack of understanding of research process, inadequate training and/or supervision, or lack of time and funding, may lead students to become disinterested in research [9, 10].

It is therefore important to explore students’ experiences of research and use their feedback to inform the future design of research projects embeded in the medical degree. This study aimed to understand medical students’ experience and satisfaction with a mandatory research project and to investigate whether students considered that the project was helpful in developing skills for future research activities. Barriers and enablers to the undertaking of these research projects were also explored.

## Context

In 2014, the Doctor of Medicine (MD) degree at The University of Sydney introduced a mandatory research project (MD research project) as part of its graduate curriculum. The students complete an independent research project under the supervision of a member of the University staff or an affiliate. Students are offered a choice of project options based, for example, on clinical, biomedical, epidemiological topics, using public health data, or based on medical education, information technology, policy, law and ethics. Students express their preferences and are matched with their research project and supervisor at the end of first year, after the delivery of the academic content on research methods and ethics. Both qualititve and quantitative research methodologies can be used for projects. The research projects are carried out in 10 centres including urban clinical schools, two rural clinical schools and the main campus of the University. Ethics approval, if required for a project, is usually obtained by the research supervisor before project commencement, although a small number of students drafted a complete research protocol (including both scientific and ethics aspects) on a major complex study as their research project. To prevent project delays or the need for extensive modification, project progress was reviewed through regular milestones. Students who did not have ethics approval in place by the third milestone (approximately six - nine months after project commencement) were assisted to rescope or amend their project to ensure that it could be completed within the available time.

The majority of project supervisors have a clinical or research background. There were no specified minimum supervisor criteria, each research hub had an MD Research Coordinator who both vetted and advised potential supervisors and provided support as needed once projects were underway. Typically, a supervisor has 2–5 students, although some have only one student. Supervisors guide students during all phases from topic selection to writing up the final report. Within their academic timetable in the first two years of their MD degree, students receive additional teaching on research methods and ethics, and sessions with librarians supporting basic literature searching. These sessions give students grounding in both qualitative and quantitative research methodologies. Progressive and final summative assessment of research projects is achieved through a series of milestone assessment tasks and the completion of a final written report, synthesising the results in a 3000 word publication format. Students have an opportunity to share their research findings through oral and poster presentations at a research symposium. During the period of this study, projects required a minimum of 320 h work over two and a half years, carried out in addition to of the overall MD program without any dedicated research time. The teaching elements of the program (e.g. lectures, workshops) and the milestone meetings are run outside of the direct research time.

## Methods

### Participants

A total of 1375 students from five cohorts who completed the medical program between 2017 and 2021 were sent the participant information sheet and link to the online survey by email after completion of their MD research project. The survey was undertaken using the Lime Survey tool (www.limesurvey.org) from 2017 to 2019 and Qualtrics^XM^ (https://www.qualtrics.com/au/ ) from 2020. Participation was voluntary, and consent was implied if the completed survey was returned. Data collection was anonymous. The study was approved by The University of Sydney Human Research Ethics Committee, (Approval #2017/748).

### Survey instrument

A survey instrument with 52 questions was developed based on extensive research of previous student evaluation literature. Content validity of the survey was determined by faculty based experts in medical education. The original draft instrument underwent pilot evaluation with students and academics/clinicians who were involved in the medical program. The reliability of the questionnaire was assessed by Cronbach’s alpha [11]. The estimated time for students to complete the survey was 30 min. A combination of questions with Likert-type responses, multiple choice answers and free text comments were used. Project-specific information was collected through seven items; information related to research skills development was collected through three items; and ten items collected information about research methods teaching. The role of research supervisors was assessed through thirteen items, and eleven items collected information about project milestones, presentations and final report writing. A final set of seven questions asked about students’ overall assessment of the project plus barriers and enablers in completing the project. Student satisfaction with the project, reported on a 10-point scale ranging from 1 for least satisfied to 10 for most satisfied, was considered the main outcome measure.

### Outcomes

Although research is frequently intended for publication, the primary aim of the MD research project is to develop important skills, such as evaluating scientific literature and understanding and experiencing the research process. The outcomes of this study were therefore measured through student self-reported research skills development, research output and overall student satisfaction with the research experience.

### Data analysis

The Cronbach’s α was found to be 0.781. Descriptive data for survey variables are presented as mean ± standard deviation of variable scores. Continuous variables were compared by independent samples t-test for two groups and analysis of variance (ANOVA) test if more than two groups were included. Categorical data were compared using Pearson’s Chi square test. A linear regression model was constructed to measure effect of various factors on student satisfaction. Variance of inflation factor to assess collinearity, standardised residuals to detect and evaluate outliers and Cook’s distance to identify influential cases were used for this model. The significance level for all tests was set at P < 0.05. Analyses were performed using IBM SPSS Statistics for Windows, Version 26.0. (IBM SPSS Statistics,Armonk, MY). Manifest content analysis, which involves describing the text instead of developing themes, [12, 13] was used to analyse textual data from open ended questions. A systematic approach known as framework analysis [14] was utilised to organise and analyse the qualitative data. The data were initially reviewed and patterns were identified, resulting in a set of codes based on the key concepts that emerged from the analysis. These codes were then applied to the data and summarised to identify any patterns or relationships between the codes. Conclusions were drawn from this analysis.

## Results

Of 1375 students invited, 577 students completed the survey, corresponding to a response rate of 42% which ranged from 40 to 43% across all five cohorts. After exclusion of incomplete surveys, a final sample of 513 was included in the analysis. Supplementary Table displays the main codes that were derived from the content analysis supported with examples quotes. These quotes are alos used in the main text of results to reinforce the relevant findings.

### Characteristics of MD research project

Most projects (n = 292, 57%) were based on clinical data, while projects based on information technology (n = 15, 3%) and medical education (n = 15, 3%) were least commonly undertaken. Almost one third (148/513, 29%) of students completed an extended rural placement either during the third or fourth year of their medical degree which meant they were remote from their project location for the final stages of the project.

### Main barriers and enablers to completing the research project

**Fig. 1:**
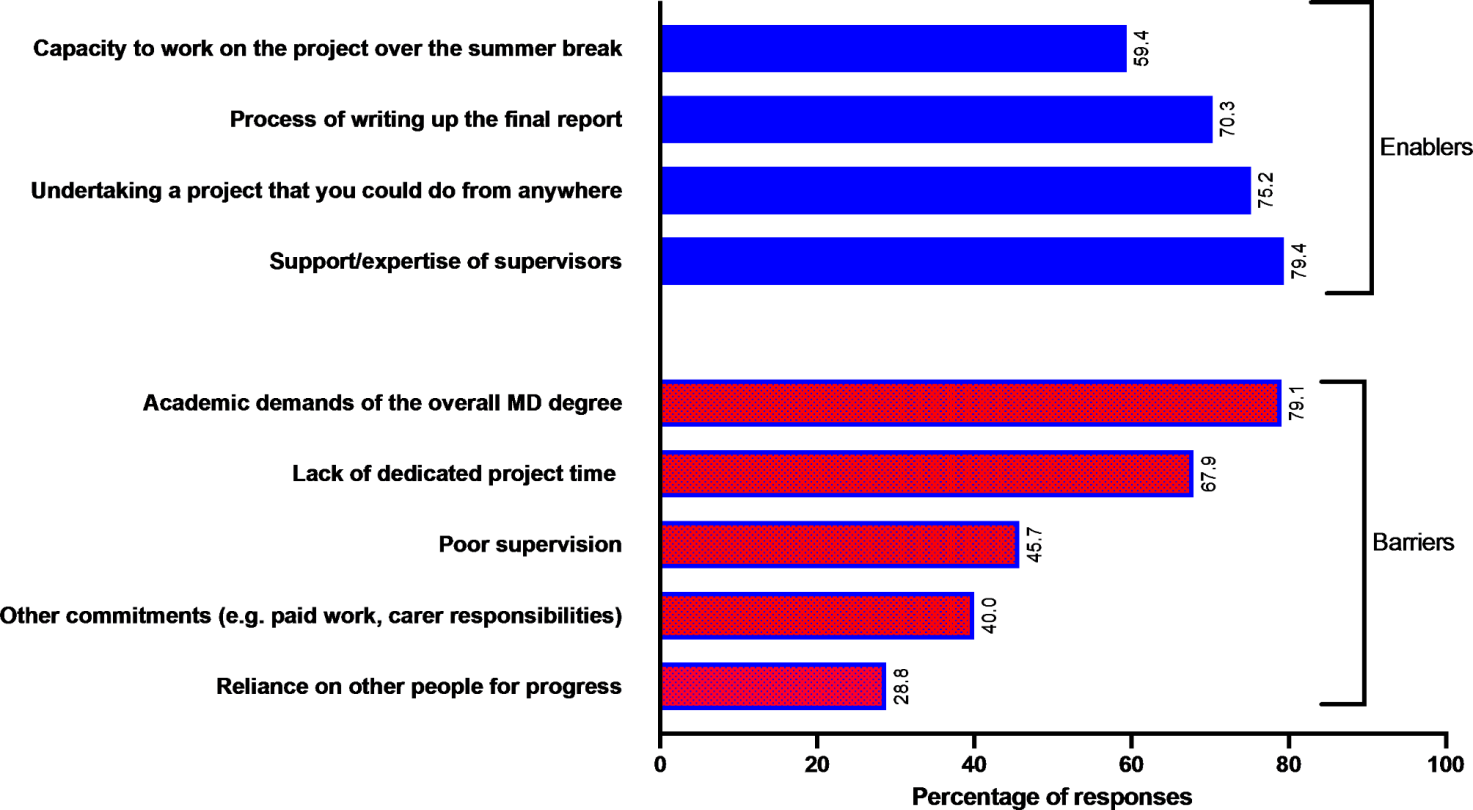
Main barriers and enablers to completing the research project. Note: Multiple responses were allowed.

Student perceptions of the main enablers to completing their research were support/expertise of the research tutor (400/513, 78%), flexibility to undertake a project they could do from different locations (364/513, 71%), and the process of writing up the final report (359/513, 70%) (See Fig. 1).

The main barriers perceived by students to completing their research project were competing academic demands of the overall medical program (379/513, 74%) and lack of dedicated time in the curriculum for the conduct of research (318/513, 62%).

### Research skills development

Only 37% (189/513) of students thought they had the necessary research skills at the beginning of their project to complete a research project. A significantly higher number of students (431/513, 84%, χ^2^ = 8.99, P = 0.02) reported having acquired the necessary research skills as a result of completing the project. Literature searching and data analysis were reported as the research skills gained by the majority of students (379/513, 74% and 426/513, 83% respectively). Responses to open ended questions overwhelmingly supported the benefits of having gained research skills, as reflected in the following quote:

*“The opportunity to independently carry out and undertake a project that improved my analytical, creative and logical skills. The project had also helped me improve my communication and critical thinking skills.”* (Student from 2021 cohort).

Some students did not feel they had acquired any new skills while a few thought it was difficult to acquire new skills, as reflected in the student quote below:

*“ I found it difficult to balance developing these skills with the other demands of the project.”* (Student from 2021 cohort).

A few students felt encouraged to undertake research in the future, as expressed in the quotes below:

*“The research project is valuable as it has given me the confidence to seek future research opportunities. Going from literally zero to a complete research project. Learning how to search the literature, develop a research question, devise methodology, collect data, analyse data, and write up a paper. A stepping stone towards what a real-life situation would be once fully graduated and furthering a career*.” (Student from 2020 cohort).

*“It was a good experience to go through the process of doing research and I learned about the challenges I will face in the future if I choose to undertake research.”* (Student from 2020 cohort).

### Prior research experience

More than half of the students (n = 297, 58%) reported that they had worked on a research project during their undergraduate or postgraduate studies prior to medical school. 38% (114/297) of these students had done an Honours project and 14% (43/297) reported having a doctoral degree, while others reported having gained research experience through a summer research project, Masters’ degree, or through a research assistant role. Most (n = 190, 64%) of these students found their prior research experience to be helpful in completing their research project.

### Utility of research skills training activities

**Fig. 2:**
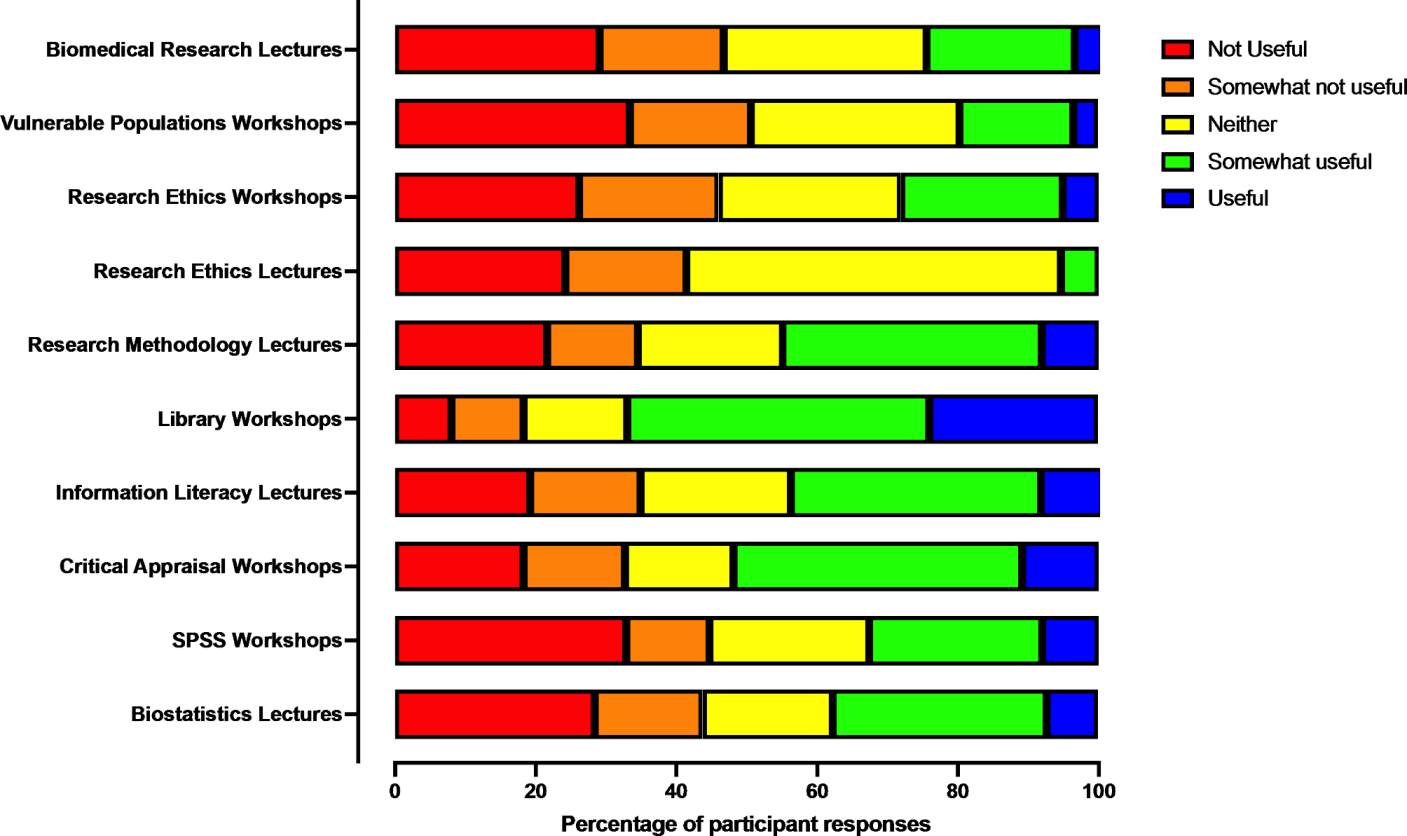
Rating of usefulness of research methods teaching in completing the research project

Only 44% (225/513) of students considered research methods teaching as helpful in relation to their capacity to complete their MD research project. Research skills teaching methods and their perceived utility are shown in Fig. 2. Library workshops, critical appraisal workshops and research methodology lectures were considered most beneficial by students.

### Role of supervisor

The majority of students considered their supervisors as supportive (353/513, 83%), good in communicating expectations (379/513,74%), and highly experienced in research (430/513,84%). Only 31% (159/513) reported meeting their supervisors regularly in addition to mandated milestone meetings, with the remaining students meeting their supervisors either infrequently or only at milestones.

The majority of students who provided feedback in open ended responses about their supervisors characterised them as exceptional, remarkable, impressive, or as a valuable asset to their research experience. An expression of positive supervisor experience was reflected in the following quote:

*“My supervisor was very enthusiastic, knowledgeable, supportive and provided an excellent learning environment and pushed me to grow in areas I was unfamiliar with.”* (Student from 2020 cohort).

Many students credited their ability to publish to their supervisors:

“*My supervisors were fantastic and made the entire MD project experience a positive one. I am grateful to have had the opportunity to work with them and to produce a piece of research which has been published-good for my CV.”* (Student from 2019 cohort).

Those who were not satisfied with their supervisor felt that either their supervsior was too busy or was inexperienced, as suggested by the following student comments:

*“ Whilst my supervisors were very nice and supportive people, they were exceptionally busy clinicians who do not have the time to fully instruct me on the steps required to conduct a research project.”* (Student from 2021 cohort).

“*My supervisor was clear in what he wanted, but did not have experience in the field so left it to other people to provide assistance. Often they were hard to to reach or provided limited assistance.”* (Student from 2019 cohort).

### Research output

21% (107/513) of students reported having submitted a manuscript based on their project to a peer reviewed journal. A further 13% (67/513) reported currently working on a manuscript for journal submission. In addition, 18% of students presented or were intending to present their findings at a local or international conference.

### Dedicated time

The need for dedicated time for completion of the research project was identified in students’ comments:

*“Having a dedicated time for it e.g., 8 weeks, rather than having to do it in between everything else over 3 years. It was nice to get into a nice flow for a few weeks then take time off due to academic demands then jump back in*” (Student from 2019 cohort).

*“A more dedicated time period for MD project completion as I found one of the most difficult parts was fitting in my MD project around my academic timetable.”* (Student from 2017 cohort).

### Student satisfaction

Overall mean satisfaction score for the MD research project was 5.0 (± 2.5) out of a total score of 10, with 59% students reporting their satisfaction above this mean score. The mean satisfaction scores ranged from 4.7 to 5.1 across five cohorts. Median score was 6 (IQR-3-7).

**Fig. 3:**
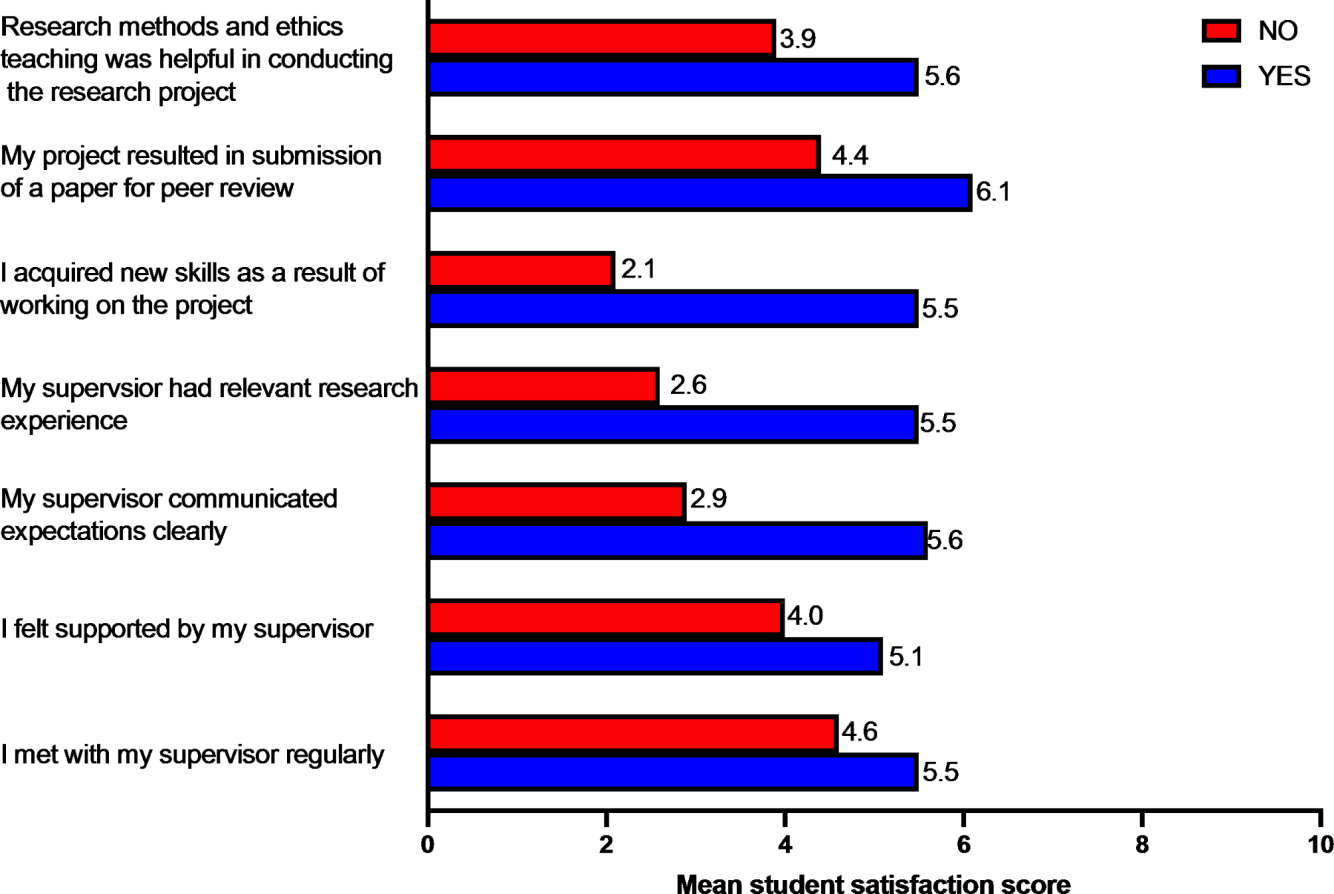
Univariate analysis of factors associated with student satisfaction. Note: P values were significant for scores comparing those who said yes from those who said no for all statements shown in the figure.

The number of students in a project group had no association with overall student satisfaction. Similarly, type of research project, project location and whether the student had prior research experience were not associated with student satisfaction. On univariate analysis significant associations were seen between student satisfaction and factors related to supervisors, perceived usefulness of research and ethics teaching, research skills development and research outputs. Mean satisfaction scores corresponding to these factors are shown in Fig. 3.

The results from linear regression model are shown in Table 1. Overall, students who reported having acquired new skills indicated a higher satisfaction score (mean = 5.5 ± 2.2) compared to those reported acquiring no new skills (mean = 2.1 ± 1.6, β = 2.937 (95% CI = 2.178–3.695; P < 0.001). Those students who considered that research methods skills and ethics teaching were helpful in gaining research skills were more satisfied (mean score = 5.6 ± 2.2) than those who did not find these to be helpful (mean score 3.9 ± 2.3, β = 1.436 (95% CI = 0.809–2.064); P < 0.001). Those who felt supported by their supervisors reportedhigher satisfaction (mean score 5.1 ± 2.3) than those who did not feel supported (mean score: 4.6 ± 2.1, β = 0.257 (95% CI=-0.119-0.633; P = 0.012). Having a research output in the form of a publication or a conference presentation led to higher mean satisfaction scores (mean score: 6.1+/-2.4) compared to not having such outputs (mean score = 4.4 ± 2.0, β = 1.014 (95% CI = 0.185–3.752;P < 0.001) (See Table 1).

**Table 1:** Results from linear regression model showing factors significantly associated with student satisfaction

Factors affecting student satisfaction	β (95% CI)	P value
The research methods ethics teaching was useful in preparing me for conducting my research.	1.436 (0.809–2.064)	< 0.001
I felt supported by my supervisor	0.257 (-0.119-0.633)	0.012
I acquired new skills as a result of working on the research project	2.937 (2.178–3.695)	< 0.001
My project resulted in a peer reviewed paper submitted for publication	1.014 (0.185–3.752	< 0.001

## Discussion

Our findings suggest that medical students considered their research experience undertaken as a mandatory research project as positive overall. Their satisfaction with the research project was mainly driven by factors such as supportive supervisors, research skills training, achieving research outputs and development of research skills.

There is a shortage of physician researchers in Australia [15]. To address this issue, it has been suggested that efforts to increase student interest in medical research should be made during the medical degree [6]. One approach to achieve this may be through an increased emphasis on research skill development during medical studies. Research projects are instructive in the principles of EBM and its relevance to clinical practice, thus helping students to understand the rationale behind their clinical decision making. A previous review established this as the main motivator in participating in research [6]. It was encouraging to find that 84% of respondents in this study reported having gained research skills.

Prior research experience has been recognised as an important motivator to undertake and complete research projects during a medical degree [16]. Similar to findings from past research [9, 16, 17] more than 50% of our students reported having research experience prior to medical school. It is expected that those with prior research experience have better knowledge, skills and confidence in conducting research than those without this experience. However, there have been mixed responses in the literature about whether students are better engaged in a research project if they have prior research experience [9]. In fact, it has been reported that medical students may not see any value in a research project to their career progression when they have a prior research based degree such as a doctorate. Our findings indicate that students who had prior research experience found it helpful to complete the research project, although their overall satisfaction scores were not different from those who did not have any prior research experience. It would be important to explore in future studies if students planned to continue research activities after medical school, and whether prior research experienced influenced this sentiment.

The role of supervisors in completing research projects and developing student appreciation of the value of research cannot be overemphasised. Supportive supervisors with relevant research experience, clear communication of expectations, and availability to meet regularly with their students were identified as the key factors contributing to higher levels of student satisfaction. It is well recognised that the success of student research initiatives rely on suitably qualified and experienced supervisors [18–20]. Our findings indicate that a supportive supervisor led to higher satisfaction with research activities. The qualitative comments in our study were overwhelmingly appreciative of supervisors. Students reported that their supervisors were knowledgeable and also guided them to develop essential research skills and, for some, to publish their work. On the other hand, poor mentorship and poor role models are among the key factors that prevent medical students from engaging in research [9, 16, 19]. Our previous research arising from the Sydney Medical Program has reported that the barriers identified by academic and clinician supervisors also include having limited research experience, and time and other resource constraints within their roles [21]. Reasons for inadequate supervision are therefore multifactorial, and our findings concur with other studies regarding the importance of the supervisor role in enabling a successful student research experience.

Our findings from this study indicate that those students who reported having published their research were more satisfied with the research project than those who did not publish. Research outputs mainly in the form of peer-reviewed publications are valued by medical students, as these are helpful in career progression and for competing and applying for future specialist training [22]. Arguably they are also considered indicators of successful research. An important skill many students learn from completing the research project is preparing their work for publication. Publishing as first author is a valuable addition to a professional *curriculum vitae* and may therefore be a contributor to future career success. However, the quality of the publication, its relevance and other factors are also likely to influence the significance of such publications. Supervisors can engage their students with research dissemination by facilitating opportunities for them to publish their research and present their findings at local and international conferences. These outcomes have the potential to foster students’ interest in research by involving them in the wider research community [23, 24].

It has also been argued that medical students should recognise the value of research for the practice of EBM and not only for gaining a competitive edge in academia and funding [25]. At the same time, research outputs are useful rewards for their accomplishments and can work as encouragements to develop their interest in research. In addition, other options to encourage medical students to undertake research should be explored, such as integrated MD/PhD degrees or sponsoring research in collaboration with other countries and universities.

### Program changes based on survey results

Addressing some of the barriers identified in current and past research may facilitate better student engagement with research. Alongside poor supervision, the main barrier identified in our study was lack of dedicated time in the timetable to do research. This barrier has been reported in other studies [9, 16, 20]. Based on evaluation feedback, commencing in 2022 The University of Sydney MD program has incorporated substantial changes to the research project, chiefly that it is now carried out in a dedicated block of 14 weeks in the third year of the program. Recognising that not all research projects can be finished within a 14-week time slot, students are given an opportunity to undertake an extended stream project over one year. Under this option students are given the choice to continue through to completion their research project alongside their other studies in addition to the dedicated 14 weeks block. The option to do an extended stream project is based on the premise that students who chose these projects may be more likely to engage in research and thus would gain higher satisfaction. Further studies could elucidate how these formats of the research project impact student satisfaction and engagement with research.

Important limitations of our study include that it is based on the experience of students at a single institution and the response rate is low. Moreover, recall bias may impact the validity of the findings. Those students who completed the survey may have been more motivated to do so because of a strongly positive or negative MD research project experience. Due to anonymity it was not possible to link survey responses to course performance as measured by summative assessment nor to assess whether the respondents were representative of the overall MD cohort during the survey period. However, the data have been collected across five cohorts and the findings are consistent across all five cohorts. An important future step would be to examine if medical students engage in further research activities beyond their degree requirements. Their involvement in research could be ascertained by examining how many of them author publications, pursue a PhD degree or apply for competitive grants and research fellowships. Our immediate next step focuses on verifying publications by screening and matching journal papers with student records. Matching this information with self-reported publication data and prior research experience would assist in confirming the robustness of findings.

In summary, our findings demonstrate that mandatory research projects during a medical degree are perceived by students as useful in developing research skills and therefore important in preparing the next generation of physician researchers to be competent adopters of evidence-based medicine. Important structural changes to the program have been made based on these study results, and further improvements will include strengthening the supportive environments for students.

## Electronic supplementary material

Below is the link to the electronic supplementary material.

Supplementary Material 1

Supplementary Material 2

## Data Availability

The datasets generated and/or analysed during the current study are not publicly available, as per conditions of Ethics Committee approval, but are available from the corresponding author on reasonable request.
