# Supplementary material for: Students’ perceived research skills development and satisfaction after completion of a mandatory research project: results from five cohorts of the Sydney medical program
Source: BMC Med Educ. 2023 Jul 12;23:502. doi: 10.1186/s12909-023-04475-y (PMC10337108; doi:10.1186/s12909-023-04475-y)
Supplement: Supplementary file 1 — Supplementary Material 1 [file 12909_2023_4475_MOESM1_ESM.docx]

Supplementary table: Framework analysis showing main codes derived from manifest content analysis of responses from open-ended questions.
