# Supplementary material for: Students’ perceived research skills development and satisfaction after completion of a mandatory research project: results from five cohorts of the Sydney medical program
Source: BMC Med Educ. 2023 Jul 12;23:502. doi: 10.1186/s12909-023-04475-y (PMC10337108; doi:10.1186/s12909-023-04475-y)
Supplement: Supplementary file 2 — Supplementary Material 2 [file 12909_2023_4475_MOESM2_ESM.docx]

| **Research Skills Development** | | | **Experiences with supervisors** | | **Need for Dedicated Project Time** |
| --- | --- | --- | --- | --- | --- |
| **New skills developed** | **No new skills developed** | **Future use of these skills** | **Positive** | **Negative** |  |
| *“The opportunity to independently carry out and undertake a project that improved my analytical, creative, and logical skills. The project had also helped me improve my communication and critical thinking skills.”*  (Student from 2021 cohort). | *“ I found it difficult to balance developing these skills with the other demands of the project.”*  *(Student from 2021 cohort).* | *“The research project is valuable as it has given me the confidence to seek future research opportunities. Going from literally zero to a complete research project. Learning how to search the literature, develop a research question, devise methodology, collect data, analyse data, and write up a paper.  A steppingstone towards what a real-life situation would be once fully graduated and furthering a career.”*  *(Student from 2020 cohort)* | “*My supervisors were fantastic and made the entire MD project experience a positive one. I am grateful to have had the opportunity to work with them and to produce a piece of research which has been published-good for my CV.”* (Student from 2019 cohort). | *“Whilst my supervisors were very nice and supportive people, they were exceptionally busy clinicians who do not have the time to fully instruct me on the steps required to conduct a research project.”* (Student from 2021 cohort) | *“Having a dedicated time for it e.g., 8 weeks, rather than having to do it in between everything else over 3 years. It was nice to get into a nice flow for a few weeks then take time off due to academic demands then jump back in*”.  (Student from 2019 cohort). |
| *“The ability to choose my own project and gain useful skills e.g. how to perform a literature search, writing skills. Excellent way to develop skills” (Student from 2020 cohort)* | *“I learned necessary skills for my narrative review, I still do not feel as though I have any skills in research outside of narrative reviews”.*  *(Student from 2019 cohort)* | *“It was a good experience to go through the process of doing research and I learned about the challenges.*  *(Student from 2020 cohort).* | *“My supervisor was very enthusiastic, knowledgeable, supportive and provided an excellent learning environment and pushed me to grow in areas I was unfamiliar with.”* (Student from 2020 cohort). | “*My supervisor was clear in what he wanted but did not have experience in the field so left it to other people to provide assistance. Often, they were hard to reach or provided limited assistance.”* (Student from 2019 cohort) | *“A more dedicated time period for MD project completion as I found one of the most difficult parts was fitting in my MD project around my academic timetable.”* (Student from 2017 cohort) |
